# Crystal structure of (*E*)-4-[*N*-(7-methyl-2-phenyl­imidazo[1,2-*a*]pyridin-3-yl)carboximido­yl]phenol

**DOI:** 10.1107/S2056989015017843

**Published:** 2015-09-30

**Authors:** Abdelmalik Elaatiaoui, Rafik Saddik, Noureddine Benchat, Mohamed Saadi, Lahcen El Ammari

**Affiliations:** a Laboratory of Applied Chemistry and Environment (LCAE), Faculty of Sciences, University Mohammed Premier, Oujda, Morocco; b Observatoire de la Lagune Marchica de Nador et Région Limitrophe, Université Mohammed Premier, Faculté Pluridisciplinaire de Nador, BP 300, Selouane 62702 Nador, Morocco; c Laboratoire de Chimie du Solide Appliquée, Faculté des Sciences, Université Mohammed V, Avenue Ibn Battouta, BP 1014, Rabat, Morocco

**Keywords:** crystal structure, imidazo[1,2*a*]pyridine derivative, hydrogen bonding, C—H⋯π inter­actions

## Abstract

The mol­ecule of the title compound, C_21_H_17_N_3_O, is built up from fused five- and six-membered rings connected to a methyl group, a phenyl ring and an (imino­meth­yl)phenol group. The fused ring system is almost planar (r.m.s. deviation = 0.031 Å) and forms dihedral angles of 64.97 (7) and 18.52 (6)° with the phenyl ring and the (imino­meth­yl)phenol group, respectively. In the crystal, centrosymmetric mol­ecules are linked by pairs of C—H⋯π inter­actions into dimeric units, which are further connected by O–H⋯N hydrogen bonds to form layers parallel to (101).

## Related literature   

For the biological activities of imidazo[1,2*a*]pyridine derivatives, see: Solomons *et al.* (1997 ▸); Bhandari *et al.* (2008 ▸); Ertl *et al.* (2000 ▸). For the synthesis of related compounds, see: Radi *et al.* (2015 ▸); Elaatiaoui *et al.* (2014 ▸).
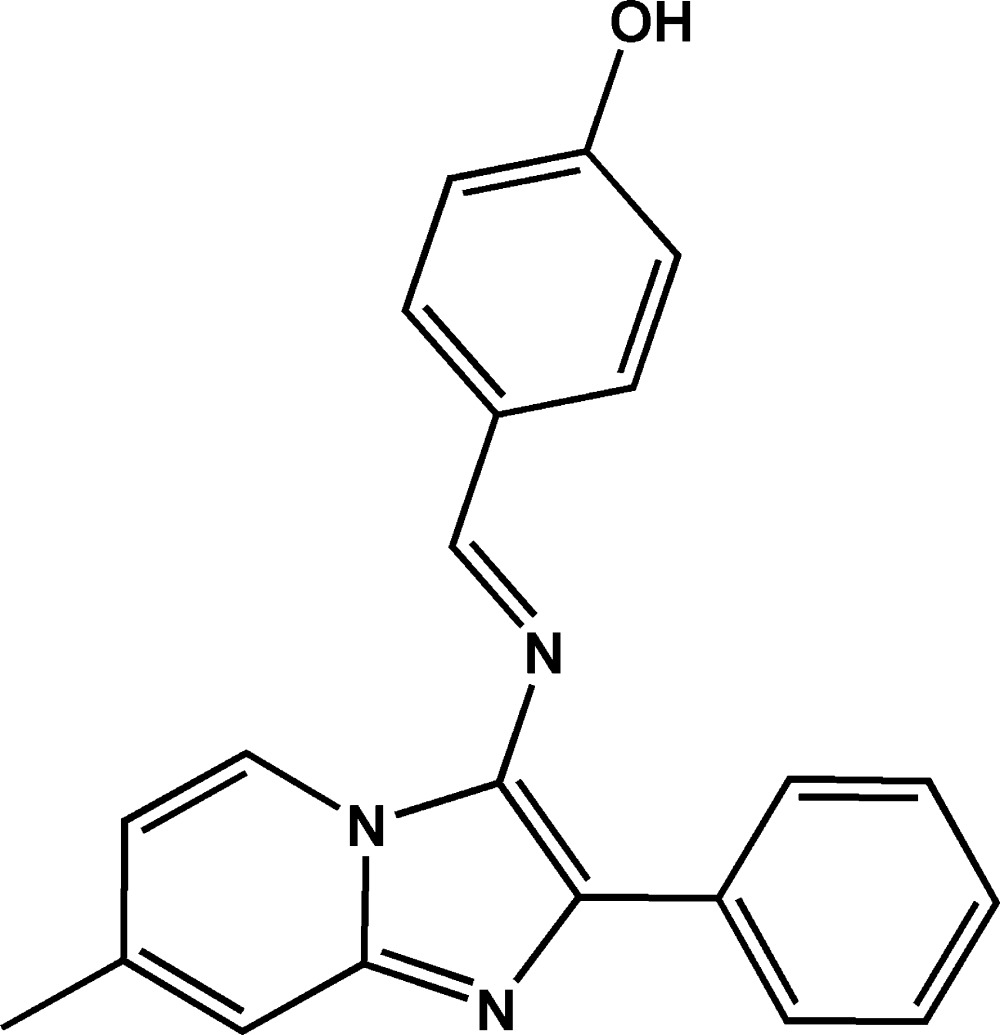


## Experimental   

### Crystal data  

C_21_H_17_N_3_O
*M* *_r_* = 327.38
Monoclinic 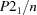

*a* = 12.295 (3) Å
*b* = 9.587 (2) Å
*c* = 14.977 (4) Å
β = 101.548 (1)°
*V* = 1729.6 (7) Å^3^
*Z* = 4
Mo *K*α radiation
μ = 0.08 mm^−1^
*T* = 296 K
0.42 × 0.31 × 0.26 mm

### Data collection  

Bruker X8 APEX Diffractometer
Absorption correction: multi-scan (*SADABS*; Bruker, 2009 ▸) *T*_min_ = 0.673, *T*_max_ = 0.746
26727 measured reflections
4126 independent reflections
2970 reflections with *I* > 2σ(*I*)
*R*_int_ = 0.033

### Refinement  

*R*[*F* ^2^ > 2σ(*F* ^2^)] = 0.045
*wR*(*F* ^2^) = 0.140
*S* = 1.02
4126 reflections
226 parameters
H-atom parameters constrained
Δρ_max_ = 0.30 e Å^−3^
Δρ_min_ = −0.18 e Å^−3^

### 

Data collection: *APEX2* (Bruker, 2009 ▸); cell refinement: *SAINT* (Bruker, 2009 ▸); data reduction: *SAINT*; program(s) used to solve structure: *SHELXS97* (Sheldrick, 2008 ▸); program(s) used to refine structure: *SHELXL2014*/7 (Sheldrick, 2015 ▸); molecular graphics: *ORTEP-3 for Windows* (Farrugia, 2012 ▸); software used to prepare material for publication: *PLATON* (Spek, 2009 ▸) and *publCIF* (Westrip, 2010 ▸).

## Supplementary Material

Crystal structure: contains datablock(s) I. DOI: 10.1107/S2056989015017843/rz5169sup1.cif

Structure factors: contains datablock(s) I. DOI: 10.1107/S2056989015017843/rz5169Isup2.hkl

**Fig. 1.**
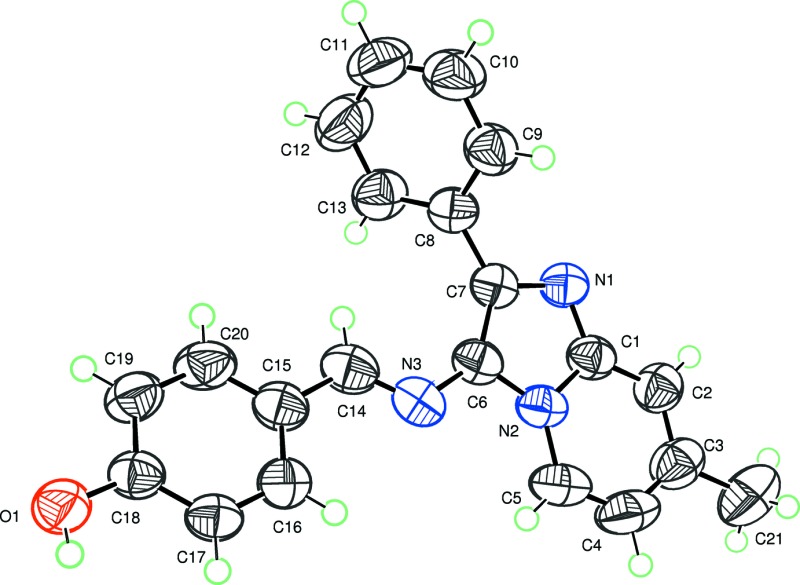
The molecular structure of the title compound with displacement ellipsoids drawn at the 50% probability level. H atoms are represented as small circles of arbitrary radius.

. DOI: 10.1107/S2056989015017843/rz5169fig1.tif
The mol­ecular structure of the title compound with displacement ellipsoids drawn at the 50% probability level. H atoms are represented as small circles of arbitrary radius.

**Fig. 2.**
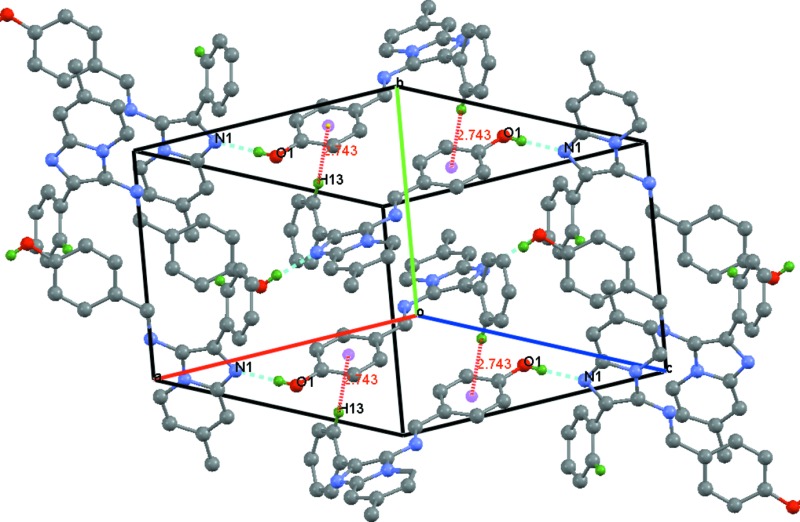
Packing diagram of the title compound showing the fornation of a layer parallel to the (1 0 1) plane by O—H···N hydrogen bonds cyan dotted lines) and C—H···π hydrogen interactions (red dotted lines). Hydrogen atoms not invoolved in hydrogen bonding are omitted.

. DOI: 10.1107/S2056989015017843/rz5169fig2.tif
Packing diagram of the title compound showing the fornation of a layer parallel to the (1 0 1) plane by O—H⋯N hydrogen bonds cyan dotted lines) and C—H⋯π hydrogen inter­actions (red dotted lines). Hydrogen atoms not invoolved in hydrogen bonding are omitted.

CCDC reference: 1426925

Additional supporting information:  crystallographic information; 3D view; checkCIF report

## Figures and Tables

**Table 1:** Hydrogen-bond geometry (, ) *Cg*1 is the centroid of the C15C20 ring.

*D*H*A*	*D*H	H*A*	*D* *A*	*D*H*A*
C13H13*Cg*1^i^	0.93	2.74	3.6705(18)	175
O1H1N1^ii^	0.82	1.86	2.6699(17)	170

Symmetry codes: (i) 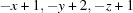; (ii) 
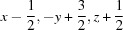
.

## supplementary crystallographic information

### S1. Comment

Our interest on imidazo[1,2a]pyridine derivatives allowed us to investigate on the synthesis with a good yield of novel Schiff base compounds from this series by using acetic acid as catalyst (Radi *et al.*, 2015). Schiff bases are known for their important therapeutic properties (Solomons *et al.*, 1997; Bhandari *et al.*, 2008; Ertl *et al.*, 2000). The present paper is a continuation of our research work devoted to the development of imidazo[1,2a]pyridine derivatives with potential pharmacological activities (Elaatiaoui *et al.*, 2014).

The molecular structure of the title compound is shown in Fig. 1. The fused five- and six-membered rings are almost coplanar, with a maximum deviation of 0.054 (2) Å for atom C4. The mean plane through the fused ring system makes dihedral angles of 64.97 (7)° and 18.52 (6)° with the phenyl ring (C8–C13) and the (iminomethyl)phenol group (N3/C14–C20), respectively. The dihedral angle between the two aromatic rings C8–C13 and C15–C20 is 69.25 (7)°. The cohesion of the crystal structure is ensured by C—H···π interactions (Table 1) linking centrosymmetrically-related molecules into dimeric units, which are further connected by O—H···N hydrogen bonds (Table 1) to form layers parallel to the (1 0 1) plane as shown in Fig. 2.

### S2. Experimental

To 7-Methyl-2-phenylimidazo[1,2-*a*]pyridin-3-amine (2.39 mmol) dissolved in 20 ml of dry diethyl ether two drops of acetic acid as catalyst (0.3 ml) were added, and the solution was stirred for 15–20 minutes at room temperature. Then 4-hydroxybenzaldehyde (2.39 mmol) was added and the reaction mixture stirred for 24 h at room temperature. The reaction was monitored by TLC. The formed product was filtered and washed with dry ether. The final purification was performed by recrystallization from hot methanol to give a crystalline powder. The powder was recrystallized from methanol and after 3 days the formed green crystals were filtered on Hotman paper (Yield 92.15%).

Spectral data. *R*_f_ = 0.50 (silica, CH2Cl2/CH3OH: 9/1). ^1^HNMR (300 MHz, DMSO, δ p.p.m.): 8.659 (s, 1H, HC18=N); 8.322 (d, 1H, C3H, J= 6.99 Hz); 7.842 (d, 2H, C12H C16H, J=7.29 Hz); 7.654 (d, 2H, C15H + C13H, J=22,2 Hz); 7.35 (q, 4H, C6H + C14H, C24H + C20H); 6.75 (d, 3H, C2H + C23H + C21H, J = 6.09 Hz); 2.41 (s, 3H, C17H). ^13^C NMR (75 MHz, DMSO, δ p.p.m.): 171.94; 160.87; 158.06; 142.28; 135.41; 134.78; 132.47; 130.38; 128.55; 128.48; 127.54; 127.26; 123.04; 115.80; 115.22; 115.02; 20.76. m/*z* (*M*+): 328.00. IR: ν (CH═N, imine) = 1655 cm^-1^; ν(OH)=3450 cm^-1^.

### S3. Refinement

H atoms were located in a difference map and treated as riding with C–H = 0.93-0.96 Å, O–H = 0.82 Å, and with *U*_iso_(H) = 1.5 *U*_eq_(C, O) for methyl and hydroxide H atoms and *U*_iso_(H) = 1.2 *U*_eq_(C) for aromatic H atoms. One reflection (1 0 1) affected by beamstop was removed during the last cycles of refinement.

### Crystal data

**Table:**

C_21_H_17_N_3_O	*F*(000) = 688
*M**_r_* = 327.38	*D*_x_ = 1.257 Mg m^−^^3^
Monoclinic, *P*2_1_/*n*	Mo *K*α radiation, λ = 0.71073 Å
*a* = 12.295 (3) Å	Cell parameters from 4126 reflections
*b* = 9.587 (2) Å	θ = 2.4–27.9°
*c* = 14.977 (4) Å	µ = 0.08 mm^−^^1^
β = 101.548 (1)°	*T* = 296 K
*V* = 1729.6 (7) Å^3^	Block, green
*Z* = 4	0.42 × 0.31 × 0.26 mm

### Data collection

**Table:**

Bruker X8 APEX Diffractometer	4126 independent reflections
Radiation source: fine-focus sealed tube	2970 reflections with *I* > 2σ(*I*)
Graphite monochromator	*R*_int_ = 0.033
φ and ω scans	θ_max_ = 27.9°, θ_min_ = 2.4°
Absorption correction: multi-scan (*SADABS*; Bruker, 2009)	*h* = −16→16
*T*_min_ = 0.673, *T*_max_ = 0.746	*k* = −12→12
26727 measured reflections	*l* = −15→19

### Refinement

**Table:**

Refinement on *F*^2^	0 restraints
Least-squares matrix: full	Hydrogen site location: inferred from neighbouring sites
*R*[*F*^2^ > 2σ(*F*^2^)] = 0.045	H-atom parameters constrained
*wR*(*F*^2^) = 0.140	*w* = 1/[σ^2^(*F*_o_^2^) + (0.0656*P*)^2^ + 0.4096*P*] where *P* = (*F*_o_^2^ + 2*F*_c_^2^)/3
*S* = 1.02	(Δ/σ)_max_ = 0.001
4126 reflections	Δρ_max_ = 0.30 e Å^−^^3^
226 parameters	Δρ_min_ = −0.18 e Å^−^^3^

### Special details

**Table:**

Geometry. All e.s.d.'s (except the e.s.d. in the dihedral angle between two l.s. planes) are estimated using the full covariance matrix. The cell e.s.d.'s are taken into account individually in the estimation of e.s.d.'s in distances, angles and torsion angles; correlations between e.s.d.'s in cell parameters are only used when they are defined by crystal symmetry. An approximate (isotropic) treatment of cell e.s.d.'s is used for estimating e.s.d.'s involving l.s. planes.

### Fractional atomic coordinates and isotropic or equivalent isotropic displacement parameters (Å^2^)

**Table:**

	*x*	*y*	*z*	*U*_iso_*/*U*_eq_	
C1	0.54952 (12)	0.49279 (15)	0.29735 (10)	0.0451 (3)	
C2	0.50833 (14)	0.40312 (17)	0.22392 (11)	0.0535 (4)	
H2	0.5552	0.3713	0.1867	0.064*	
C3	0.39967 (15)	0.36297 (19)	0.20743 (12)	0.0615 (4)	
C4	0.33099 (15)	0.4141 (2)	0.26548 (14)	0.0695 (5)	
H4	0.2572	0.3858	0.2555	0.083*	
C5	0.36876 (13)	0.5026 (2)	0.33486 (13)	0.0620 (5)	
H5	0.3219	0.5349	0.3719	0.074*	
C6	0.53862 (12)	0.62960 (16)	0.41673 (10)	0.0452 (3)	
C7	0.64653 (12)	0.62297 (15)	0.40304 (9)	0.0418 (3)	
C8	0.75103 (12)	0.68006 (16)	0.45771 (9)	0.0436 (3)	
C9	0.83189 (14)	0.58748 (19)	0.50032 (11)	0.0561 (4)	
H9	0.8193	0.4920	0.4940	0.067*	
C10	0.93079 (16)	0.6352 (2)	0.55190 (12)	0.0696 (5)	
H10	0.9839	0.5719	0.5805	0.083*	
C11	0.95080 (16)	0.7758 (3)	0.56102 (14)	0.0751 (6)	
H11	1.0170	0.8079	0.5964	0.090*	
C12	0.87302 (17)	0.8688 (2)	0.51789 (14)	0.0727 (5)	
H12	0.8872	0.9641	0.5232	0.087*	
C13	0.77345 (14)	0.82168 (18)	0.46638 (12)	0.0579 (4)	
H13	0.7212	0.8856	0.4374	0.069*	
C14	0.52384 (14)	0.78868 (19)	0.52891 (12)	0.0562 (4)	
H14	0.5941	0.8193	0.5237	0.067*	
C15	0.47106 (12)	0.85783 (17)	0.59525 (10)	0.0484 (4)	
C16	0.36939 (12)	0.81542 (16)	0.61444 (10)	0.0470 (3)	
H16	0.3318	0.7408	0.5826	0.056*	
C17	0.32421 (12)	0.88222 (16)	0.67956 (10)	0.0464 (3)	
H17	0.2574	0.8511	0.6924	0.056*	
C18	0.37814 (12)	0.99643 (16)	0.72644 (10)	0.0457 (3)	
C19	0.47875 (13)	1.04114 (19)	0.70700 (11)	0.0556 (4)	
H19	0.5152	1.1177	0.7373	0.067*	
C20	0.52369 (13)	0.9715 (2)	0.64294 (11)	0.0586 (4)	
H20	0.5914	1.0013	0.6311	0.070*	
C21	0.3546 (2)	0.2628 (2)	0.13212 (16)	0.0885 (7)	
H21A	0.2772	0.2469	0.1308	0.133*	
H21B	0.3636	0.3014	0.0749	0.133*	
H21C	0.3942	0.1761	0.1425	0.133*	
N1	0.65214 (10)	0.53861 (13)	0.32982 (8)	0.0456 (3)	
N2	0.47802 (10)	0.54401 (13)	0.34959 (8)	0.0465 (3)	
N3	0.48107 (11)	0.68926 (14)	0.47735 (9)	0.0506 (3)	
O1	0.33670 (9)	1.06880 (13)	0.78932 (7)	0.0589 (3)	
H1	0.2777	1.0339	0.7953	0.088*	

### Atomic displacement parameters (Å^2^)

**Table:**

	*U*^11^	*U*^22^	*U*^33^	*U*^12^	*U*^13^	*U*^23^
C1	0.0404 (7)	0.0465 (8)	0.0483 (8)	0.0019 (6)	0.0084 (6)	0.0070 (6)
C2	0.0550 (9)	0.0524 (9)	0.0513 (9)	−0.0014 (7)	0.0063 (7)	0.0013 (7)
C3	0.0577 (10)	0.0605 (10)	0.0600 (10)	−0.0065 (8)	−0.0028 (8)	0.0048 (8)
C4	0.0408 (9)	0.0817 (13)	0.0793 (13)	−0.0094 (9)	−0.0039 (8)	0.0051 (10)
C5	0.0361 (8)	0.0752 (11)	0.0742 (11)	0.0034 (8)	0.0096 (8)	0.0066 (9)
C6	0.0412 (8)	0.0482 (8)	0.0463 (8)	0.0044 (6)	0.0095 (6)	0.0059 (6)
C7	0.0411 (7)	0.0428 (7)	0.0422 (7)	0.0013 (6)	0.0096 (6)	0.0064 (6)
C8	0.0408 (7)	0.0510 (8)	0.0399 (7)	−0.0002 (6)	0.0106 (6)	0.0044 (6)
C9	0.0544 (9)	0.0591 (9)	0.0527 (9)	0.0081 (8)	0.0053 (7)	0.0004 (7)
C10	0.0537 (10)	0.0908 (14)	0.0582 (10)	0.0154 (10)	−0.0031 (8)	−0.0059 (10)
C11	0.0495 (10)	0.1046 (16)	0.0673 (12)	−0.0103 (11)	0.0020 (8)	−0.0204 (11)
C12	0.0654 (12)	0.0690 (12)	0.0826 (13)	−0.0206 (10)	0.0119 (10)	−0.0095 (10)
C13	0.0540 (9)	0.0538 (9)	0.0642 (10)	−0.0036 (8)	0.0078 (8)	0.0086 (8)
C14	0.0481 (9)	0.0665 (10)	0.0577 (9)	0.0027 (8)	0.0193 (7)	0.0031 (8)
C15	0.0444 (8)	0.0551 (9)	0.0469 (8)	0.0026 (7)	0.0121 (6)	0.0052 (7)
C16	0.0457 (8)	0.0453 (8)	0.0497 (8)	0.0000 (6)	0.0092 (6)	0.0052 (6)
C17	0.0381 (7)	0.0524 (8)	0.0500 (8)	−0.0015 (6)	0.0118 (6)	0.0073 (7)
C18	0.0406 (8)	0.0560 (8)	0.0403 (7)	0.0008 (6)	0.0077 (6)	0.0037 (6)
C19	0.0467 (8)	0.0688 (10)	0.0520 (9)	−0.0129 (8)	0.0116 (7)	−0.0071 (8)
C20	0.0435 (8)	0.0778 (11)	0.0576 (9)	−0.0110 (8)	0.0180 (7)	−0.0022 (8)
C21	0.0867 (15)	0.0875 (15)	0.0824 (14)	−0.0259 (12)	−0.0044 (12)	−0.0115 (12)
N1	0.0410 (6)	0.0489 (7)	0.0476 (7)	−0.0011 (5)	0.0108 (5)	0.0019 (5)
N2	0.0366 (6)	0.0509 (7)	0.0513 (7)	0.0028 (5)	0.0068 (5)	0.0054 (6)
N3	0.0474 (7)	0.0553 (8)	0.0508 (7)	0.0083 (6)	0.0139 (6)	0.0045 (6)
O1	0.0498 (6)	0.0726 (8)	0.0577 (7)	−0.0102 (5)	0.0184 (5)	−0.0137 (6)

### Geometric parameters (Å, º)

**Table:**

C1—N1	1.3326 (19)	C11—H11	0.9300
C1—N2	1.3789 (19)	C12—C13	1.386 (3)
C1—C2	1.408 (2)	C12—H12	0.9300
C2—C3	1.364 (2)	C13—H13	0.9300
C2—H2	0.9300	C14—N3	1.272 (2)
C3—C4	1.415 (3)	C14—C15	1.451 (2)
C3—C21	1.500 (3)	C14—H14	0.9300
C4—C5	1.350 (3)	C15—C20	1.390 (2)
C4—H4	0.9300	C15—C16	1.398 (2)
C5—N2	1.375 (2)	C16—C17	1.374 (2)
C5—H5	0.9300	C16—H16	0.9300
C6—N3	1.3818 (19)	C17—C18	1.395 (2)
C6—C7	1.384 (2)	C17—H17	0.9300
C6—N2	1.393 (2)	C18—O1	1.3491 (18)
C7—N1	1.3753 (19)	C18—C19	1.394 (2)
C7—C8	1.483 (2)	C19—C20	1.372 (2)
C8—C13	1.386 (2)	C19—H19	0.9300
C8—C9	1.389 (2)	C20—H20	0.9300
C9—C10	1.382 (2)	C21—H21A	0.9600
C9—H9	0.9300	C21—H21B	0.9600
C10—C11	1.372 (3)	C21—H21C	0.9600
C10—H10	0.9300	O1—H1	0.8200
C11—C12	1.371 (3)		
			
N1—C1—N2	109.89 (13)	C12—C13—C8	120.56 (17)
N1—C1—C2	130.74 (14)	C12—C13—H13	119.7
N2—C1—C2	119.36 (14)	C8—C13—H13	119.7
C3—C2—C1	119.98 (16)	N3—C14—C15	124.79 (16)
C3—C2—H2	120.0	N3—C14—H14	117.6
C1—C2—H2	120.0	C15—C14—H14	117.6
C2—C3—C4	118.30 (16)	C20—C15—C16	117.76 (14)
C2—C3—C21	121.16 (19)	C20—C15—C14	118.96 (14)
C4—C3—C21	120.50 (18)	C16—C15—C14	123.28 (15)
C5—C4—C3	122.34 (16)	C17—C16—C15	121.05 (15)
C5—C4—H4	118.8	C17—C16—H16	119.5
C3—C4—H4	118.8	C15—C16—H16	119.5
C4—C5—N2	118.64 (17)	C16—C17—C18	120.29 (14)
C4—C5—H5	120.7	C16—C17—H17	119.9
N2—C5—H5	120.7	C18—C17—H17	119.9
N3—C6—C7	138.47 (15)	O1—C18—C19	117.56 (14)
N3—C6—N2	116.61 (13)	O1—C18—C17	123.18 (13)
C7—C6—N2	104.84 (12)	C19—C18—C17	119.25 (14)
N1—C7—C6	110.35 (13)	C20—C19—C18	119.66 (16)
N1—C7—C8	118.76 (12)	C20—C19—H19	120.2
C6—C7—C8	130.65 (13)	C18—C19—H19	120.2
C13—C8—C9	118.18 (15)	C19—C20—C15	121.97 (15)
C13—C8—C7	123.17 (14)	C19—C20—H20	119.0
C9—C8—C7	118.62 (14)	C15—C20—H20	119.0
C10—C9—C8	120.95 (17)	C3—C21—H21A	109.5
C10—C9—H9	119.5	C3—C21—H21B	109.5
C8—C9—H9	119.5	H21A—C21—H21B	109.5
C11—C10—C9	120.06 (18)	C3—C21—H21C	109.5
C11—C10—H10	120.0	H21A—C21—H21C	109.5
C9—C10—H10	120.0	H21B—C21—H21C	109.5
C12—C11—C10	119.85 (18)	C1—N1—C7	106.85 (12)
C12—C11—H11	120.1	C5—N2—C1	121.28 (14)
C10—C11—H11	120.1	C5—N2—C6	130.54 (14)
C11—C12—C13	120.37 (19)	C1—N2—C6	108.05 (12)
C11—C12—H12	119.8	C14—N3—C6	120.33 (14)
C13—C12—H12	119.8	C18—O1—H1	109.5

### Hydrogen-bond geometry (Å, º)

Cg1 is the centroid of the C15–C20 ring.

**Table:**

*D*—H···*A*	*D*—H	H···*A*	*D*···*A*	*D*—H···*A*
C13—H13···*Cg*1^i^	0.93	2.74	3.6705 (18)	175
O1—H1···N1^ii^	0.82	1.86	2.6699 (17)	170

Symmetry codes: (i) −*x*+1, −*y*+2, −*z*+1; (ii) *x*−1/2, −*y*+3/2, *z*+1/2.
