# Supplementary figures and images for: Crystal structure of (E)-4-[N-(7-methyl-2-phenyl­imidazo[1,2-a]pyridin-3-yl)carboximido­yl]phenol
Source: Acta Crystallogr E Crystallogr Commun. 2015 Sep 30;71(Pt 10):o803–4. doi: 10.1107/S2056989015017843 (PMC4647394; doi:10.1107/S2056989015017843)

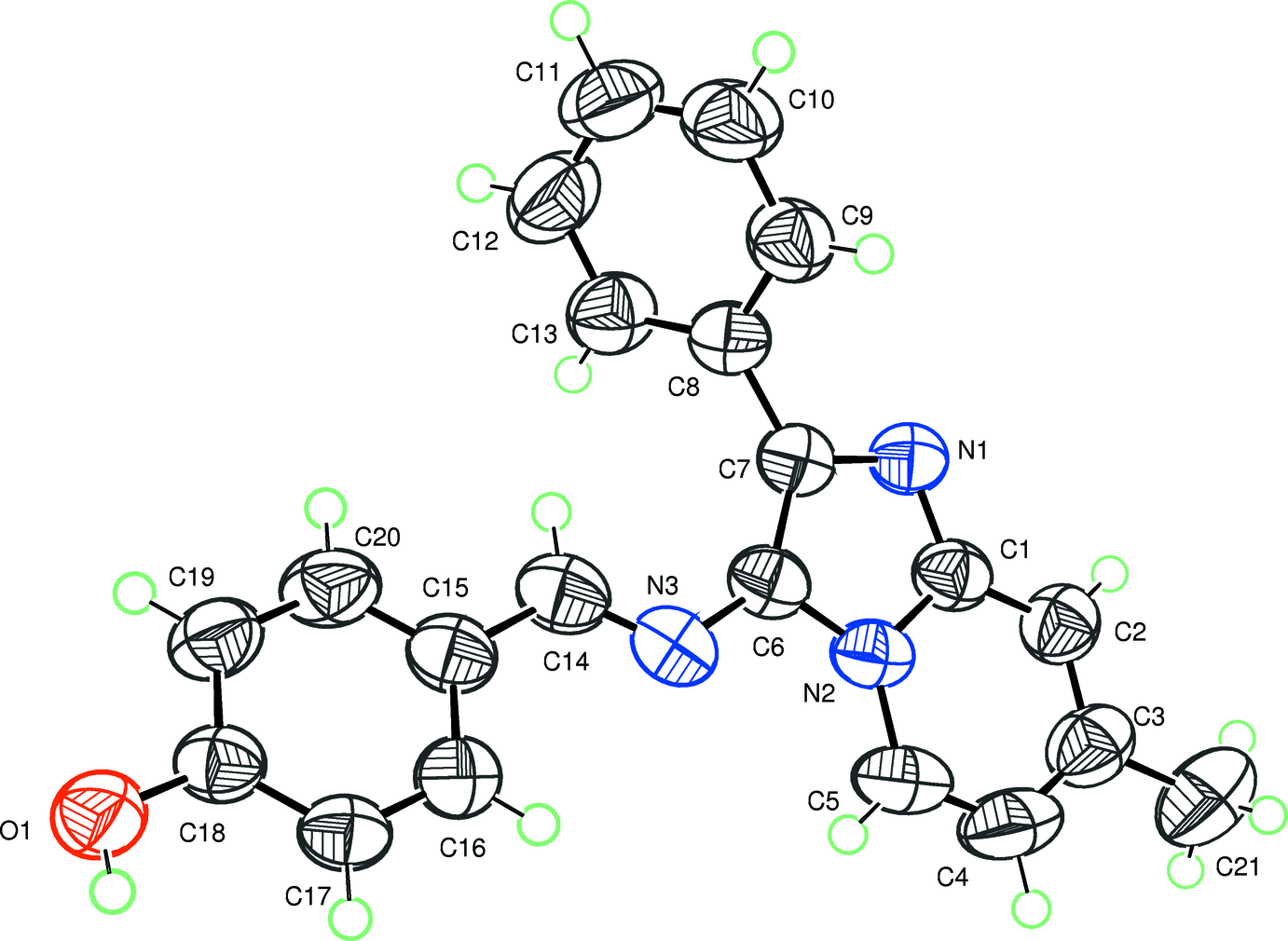

Supplement: Supplementary file 3 [file e-71-0o803-fig1.tif]

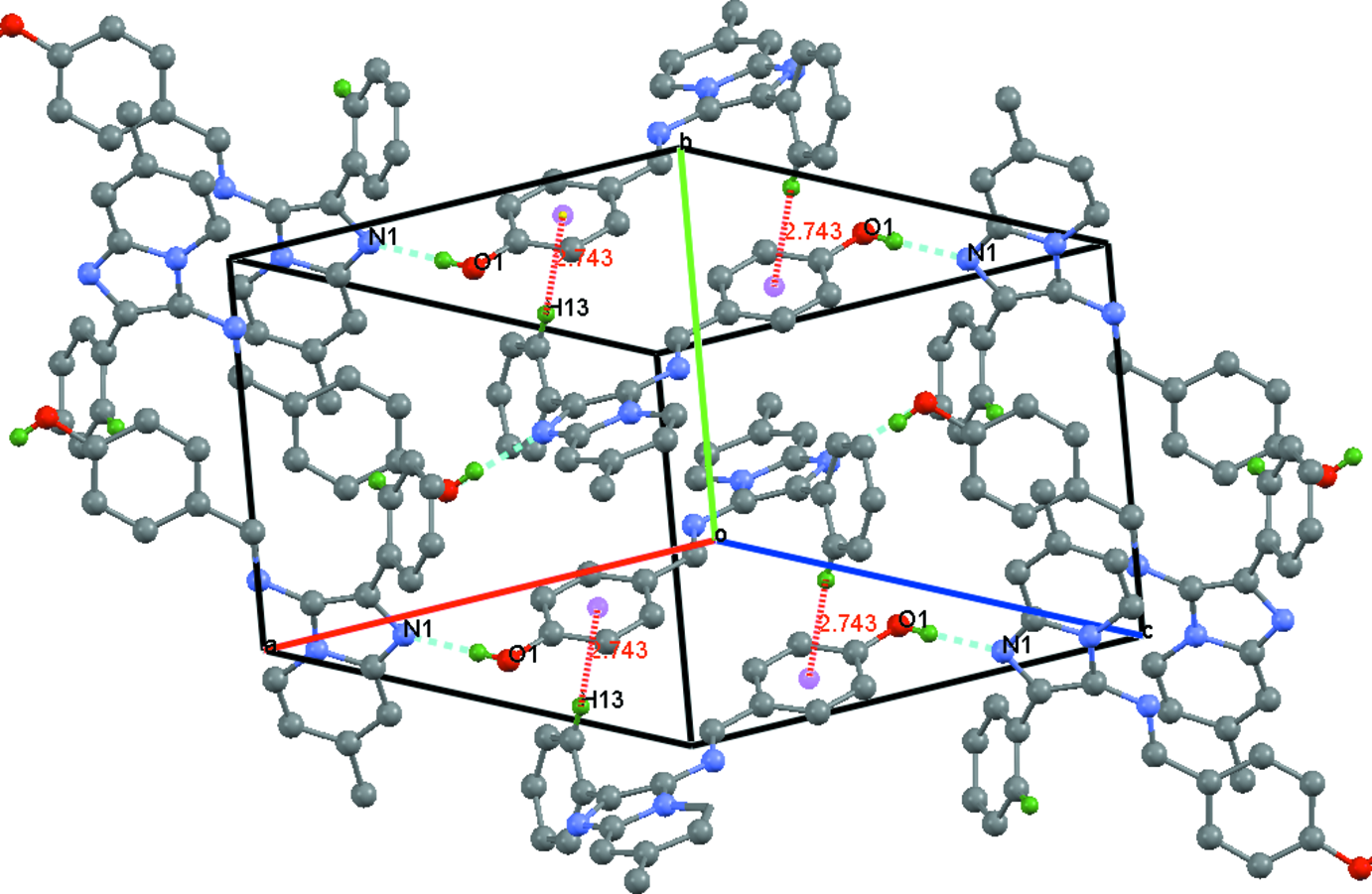

Supplement: Supplementary file 4 [file e-71-0o803-fig2.tif]
